# Application of Ultrasound Images Texture Analysis for the Estimation of Intramuscular Fat Content in the *Longissimus Thoracis* Muscle of Beef Cattle after Slaughter: A Methodological Study

**DOI:** 10.3390/ani11041117

**Published:** 2021-04-13

**Authors:** Giorgia Fabbri, Matteo Gianesella, Luigi Gallo, Massimo Morgante, Barbara Contiero, Michele Muraro, Matteo Boso, Enrico Fiore

**Affiliations:** 1 Department of Animal Medicine, Productions and Health (MAPS), University of Padua, Viale dell’Università 16, 35020 Legnaro, Italy; matteo.gianesella@unipd.it (M.G.); massimo.morgante@unipd.it (M.M.); barbara.contiero@unipd.it (B.C.); enrico.fiore@unipd.it (E.F.); 2 Department of Agronomy, Food, Natural Resources, Animals and Environment (DAFNAE), University of Padua, Viale dell’Università 16, 35020 Legnaro, Italy; luigi.gallo@unipd.it; 3 Veterinary Freelance, 37100 Verona, Italy; lelemuraro@gmail.com; 4 Veterinary Service of Società Agricola Vio, 30020 Eraclea, Italy; bosomatteo@yahoo.it

**Keywords:** beef cattle, intramuscular fat prediction, ultrasound texture analysis, carcasses IMF evaluation, ultrasonography

## Abstract

**Simple Summary:**

Fat content in the muscle mass (IMF) is one of the most important characteristics influencing the aroma, tenderness, and juiciness of the meat and therefore has high importance for both commercialization purposes and consumers. However, IMF determination currently relies on visual inspection, which is a subjective and inconsistent technique. The aim of the present study is the elaboration of a procedure capable of predicting IMF% in beef carcasses using ultrasound imaging texture analysis. Ultrasound images taken on meat samples were compared to meat composition measured by chemical extraction. Determination coefficient between the two techniques was R^2^ = 0.76, while Receiver Operating Characteristic analysis showed a sensitivity of 88% and a specificity of 90%. The results therefore suggest that the described procedure is expected to determine IMF% in muscle with good accuracy. Ultrasound imaging could be applied in routine beef grading practices. This may help to solve the issues related to subjectivity and leave to the operator only imaging acquisition. Better consistency in beef products could enhance consumers’ satisfaction and commercial standardization programs.

**Abstract:**

Intramuscular fat (IMF) is a major trait in the evaluation of beef meat, but its determination is subjective and inconsistent and still relies on visual inspection. This research objective was a method to predict IMF% from beef meat using ultrasound (US) imaging texture analysis. US images were performed on the *longissimus thoracis* muscle of 27 Charolaise heifers. Cuts from the 12th to 13th ribs were scanned. The lipid content of the muscle samples was determined with the petrol ether (Randall) extraction method. A stepwise linear discriminant analysis was used to screen US texture parameters. IMF% measured by chemical extraction (IMFqa) was the dependent variable and the results of the texture analysis were the explanatory variables. The model highlighted seven parameters, as a predictive and a multiple regression equation was created. Prediction of IMF content (IMFpred) was then validated using IMFqa as ground truth. Determination coefficient between IMFqa and IMFpred was R^2^ = 0.76, while the ROC analysis showing a sensitivity of 88% and a specificity of 90%. Bland-Altman plot upper and lower limit were +1.34 and −1.42, respectively (±1.96 SD), with a mean of −0.04. The results from the present study therefore suggest that prediction of IMF content in muscle mass by US texture analysis is possible.

## 1. Introduction

While modern trends and fashions in the food market could suggest a decrease in meat consumption, favoring vegetable-based alternatives, many studies have indicated that meat demand has increased globally and is likely to continue in the future [1]. 

One of the main challenges in the market of beef, along with ensuring food safety, is the commercialization of a product that is both homogeneous and enjoyable. Among the most important characteristics exerting a significant influence on the aroma, tenderness, and juiciness of the meat, is the fat content in the muscle mass [2,3]. Lipid content in the muscle (commonly referred as Intramuscular Fat, IMF) not only directly influences palatability traits, but also the profits, since it is often considered as directly affecting consumers’ consumption decisions [4].

Chemical analysis is still the most precise method to determine carcass composition and IMF content. It can be performed either by ether-extraction or with other fat extraction methods such as chloroform/methanol. Ether-extraction is considered to be the gold standard method for fat extraction [3,5]. However, costs, product losses, and length of time make these techniques nonviable routinely in the beef industry [4]. The preferred method to determine IMF content is marbling scoring, and the grading carcasses still relays on the visual appraisal by trained evaluators [6]. However, visual grading is an imprecise technique: it’s inconsistent and subject to the bias of the inspector (human factor) [7,8]. 

The beef industry is moving toward a value-based marketing system. The assigned conformation and fatness classification can have a heavy influence on the final economic value of a carcass [6]. There is a need for an instrumental grading system that can determine IMF% precisely, rapidly, and accurately. This is for both commercialization purposes and for consumers.

Several instrumental techniques have been tested to grade marbling with the aim of being rapid and reliable. All such techniques were very promising, but nothing has been identified as decisive yet [4,5,9,10,11].

Ultrasound (US) imaging is a very popular tool in the cattle industry, with a wide range of applications due to its ease of use. Its usefulness is not only for the reproduction sphere, but also in a variety of different clinical and research settings. These include estimation of IMF in live cattle, fatty liver evaluations, and assessing cardiovascular diseases and claw disorders [12,13,14,15]. However, although owning its popularity in the live animals IMF estimation to its acceptable degree of accuracy and easiness of use [6], there are few studies on the application of this technique on beef carcasses [6,16].

Therefore, the aim of the present study was the application of US imaging and texture analysis for the estimation of IMF% from portions of *longissimus thoracis* muscle mass, by development and testing of a prediction equation, which may be used to predict the IMF meat content.

## 2. Materials and Methods

### 2.1. Experimental Design

This study is part of the European Social Fund (ESF) project (2105-113-2121-201). 

The current research was performed on 27 Charolaise heifers’ carcasses. The animals were provided by a beef-fattening herd, located in the north-eastern part of Italy (Eraclea, Italy- 45°35′ N; 12°41′ E). Heifers were kept on a concrete slatted floor and provided a total mixed ration (Table 1) twice per day, based on 10% feed refusal (as-fed basis), and water *ad libitum* for the 2 months preceding slaughter. During this period, average daily feed intake (18 ± 1.5 kg) and dry matter intake (9.78 ± 0.8 kg) were recorded.

All the animals were slaughtered according to EU regulations (Council Regulation (EC) No 1099/2009 of 24 September 2009 on the protection of animals at the time of killing) when the fattening cycle was concluded. The fattening cycle was, on average, 7 months, after the animals arrived from France. Average age at slaughter and cold carcass weight was 17 months ± 1.8 and 362 kg ± 59 kg.

One meat sample from each carcass was collected within 6 h from slaughter. The investigated region was a section of the *longissimus thoracis* muscle (LT) collected between from the 12th to 13th ribs [15,17,18].

### 2.2. Ultrasonographic and Chemical Evaluation of the Meat Samples

**Figure 1:**
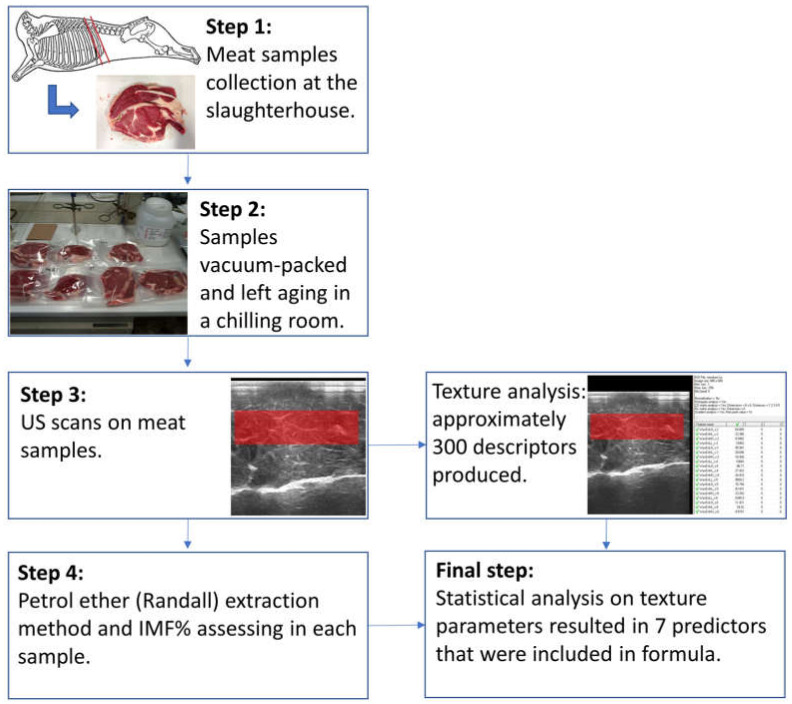
Operational flowchart of methods in the study.

A flow chart summarizing the methodological steps of the present study is depicted in Figure 1.

At the University of Padova (Laboratory of Chemistry, La-Chi, Department DAFNAE), the samples were vacuum-packed and left aging in a chilling room (4 °C) for 7 days. Before US imaging, samples were left to defreeze for 1 h at ambient temperature (22–25 °C). A portable ultrasound scanner (MyLabOne™, Esaote S.p.a., Genoa, Italy) equipped with a multi-frequency linear probe (SC3421, Esaote S.p.a., Genova, Italy; 7.5–10.0 MHz) was used to scan the LT muscle. All scans were performed with constant ultrasound settings frequency of 10.0 MHz, 7 cm depth acoustics window, 100% grey scale gain, time gain compensation was in a neutral position. For each LT muscle sample, two US scans were acquired. Images were saved in a digital imaging and communications in medicine (DICOM) format, without compression and in an 8-bit grey scale.

Centesimal extraction was performed on LT muscle samples according to Boccard, et al. (1981) [19] guidelines. Petrol ether (Randall) extraction method SOXTET 255 FOSS TECATOR (method 991.36; AOAC 2003) was used to assess IMF% [20].

### 2.3. Ultrasonographic Features Extraction and Selection

One US scan was selected for each LT muscle sample. Images were selected based on the best ultrasonic appearance (clear imaging, free of artifacts, and no shadows from costal bone residue) [17]. The scans underwent no pre-processing and texture analysis was performed using a free purpose-specific software (MaZda v4.6; Technical University of Lodz, Institute of Electronics, Poland). A rectangular region of interest (ROI) was randomly selected. The ROI was a rectangle of about 33,952.15 pixels (±15,146.17 SD).

Texture analysis produced approximately 300 descriptors, which fall into 6 main categories. Detailed description of the 6 categories (histogram features, autoregressive models, co-occurrence matrix, gradient features, run-length matrix, wavelet transform) can be found in Wu, et al. [21], Banzato, et al. [22], and the MaZda manual [23].

Ultrasound texture analysis was performed following indications from the software developers (co-occurrence matrix: 6 bits/pixel, gradient features: 8 bits/pixel, run-length matrix: 4 bits/pixel, wavelet transform: 12 bits/pixel).

### 2.4. Statistical Analysis

Statistics were performed using two statistical softwares: SAS 9.4 (SAS Inst. Inc., Cary, NC, USA) and MedCalc (MedCalc Software, Ostend, Belgium).

Animals were divided into 3 groups for statistical analysis purposes. Groups were created depending on the mean lipid content percentage in 100 g meat, and cutoffs between the groups were chosen using IMF mean ± ½SD. The three resulting groups were: Group 1: IMF ≤ 4.24%; Group 2: 4.25% ≤ IMF ≤ 5.75%; and Group 3: IMF ≥ 5.76%.

Texture parameters were tested with stepwise linear regression analysis. The aim was to identify the best combinations of high-quality predictors with as fewer parameters as possible, to improve stability during validation. IMF measured by chemical extraction (“quantified IMF”: IMFqa) served as the dependent variable, while the results of the texture analysis as explanatory variables. The regression equation that could maintain the highest number of variables, keeping the variance inflation factor lower than 10 for all included variables, was assumed as a predictive model.

The hypotheses on the linear model were graphically assessed on the residuals. The model was tested with a receiver operating characteristic (ROC) curve, and the area under the curve, and the sensitivity and specificity were evaluated. The aim of the ROC analysis was to identify the cut-off on the predictive variables that best discriminate between two samples of animals (Groups1 vs. Groups2 + Groups3). The Yuden criterion was adopted. Procedure was then validated with a Bland-Altman analysis and the agreement between IMFqa and the predicted IMF (IMFpred) verified.

Due to the not normally distribution, texture parameters, selected as most predictive for IMFqa, was analyzed in function of the three groups using a non-parametric approach (Kruskal-Wallis H test) with a Bonferroni correction of post-hoc pairwise comparisons.

## 3. Results

The results from the stepwise analysis indicated seven variables, among the 300 descriptors, as predictive. The seven texture parameters found to be significant were the following: GrKurtosis, Teta2, Teta4, S(2,2)InvDfMom, S(3,−3)Contrast, S(4,−4)DifEntrp, and 45dgr_ShrtREmp. GrKurtosis falls in the gradient category. Features from this category numerically describe the gradient values of the pixel intensities across the region of interest using a 3 × 3-pixel interval. Teta2 and Teta4 refer to the autoregressive model category. These features describe presence of a distribution pattern or random scatter of signal intensity in the ROI. S(2,2)InvDfMom, S(3,−3)Contrast, and S(4,−4)DifEntrp pertain to the Co-occurrence matrix category. These parameters provide a description of homogeneity in the ROI by analyzing the changes in pixel intensity at increasing pixel distances. 45dgr_ShrtREmp is part of the Run-length matrix category and provides a numerical description of the homogeneity of the signal intensity in specific directions of the ROI [22]. These seven variables were combined into a model that resulted in the following regression equation:

IMFpred = 281.89031 + (−208.07129 * S(2,2)InvDfMom) + (0.14783 * S(3,−3)Contrast) + (−85.60868 * S(4,−4)DifEntrp) + (−180.22176 * 45dgr_ShrtREmp) + (1.11743 * GrKurtosis) + (−44.34882 * Teta2) + (49.86069 * Teta4).(1)

The results of Kruskal-Wallis’ H test on the selected texture parameters in the function of the three Groups are reported in Table 2.

Mean IMFqa was 5.10 ± 1.44% (minimum value: 2.97%; maximum value: 8.64%), while mean IMFpred was 5.13 ± 1.31% (minimum value: 2.57%; maximum value: 7.71%). Descriptive statistics of extracted and predicted lipid content among the groups are summarized in Table 3.

**Figure 2:**
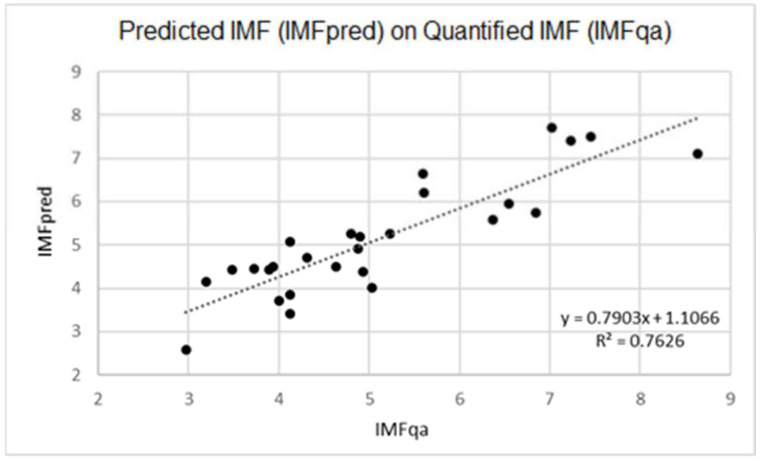
Scatterplot comparison of predicted IMF (IMFpred) and quantified IMF (IMFqa).

The difference between IMFqa and IMFpred means was −0.04 (±0.70 SD), with a determination coefficient of R^2^ = 0.76. Scatterplot in Figure 2 graphically explains the correspondence between IMFqa and IMFpred where a linear correlation was evident (R^2^ = 0.76).

**Figure 3:**
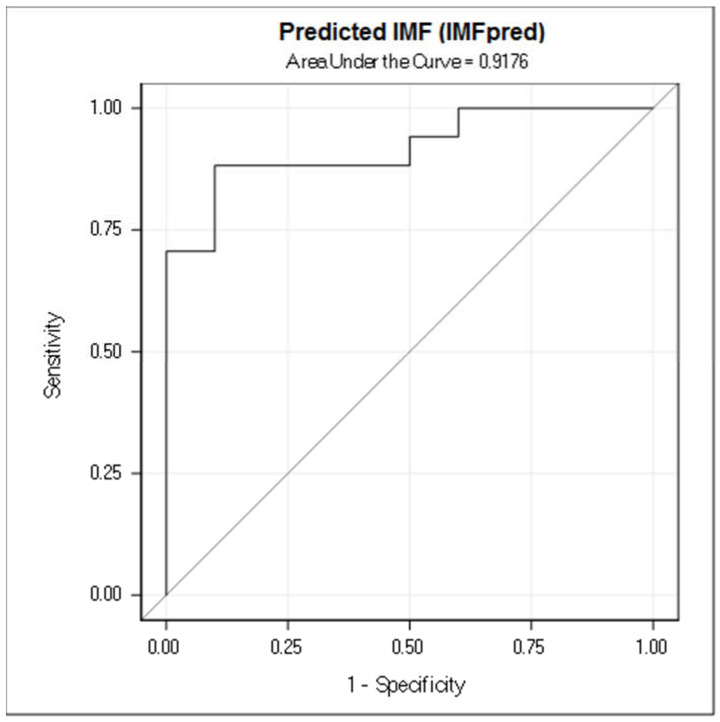
Results of the receiver operator curve (ROC) for the samples. Area Under the Curve = 0.9176 (95% CI: 0.8141–1.0000; positive likelihood ratio 16.9749) using an optimal cut-off value of 4.50 IMF% sensitivity was 88% and the specificity was 90%.

Figure 3 illustrates the results from the ROC analysis, describing the estimation of lipid content percentage in the muscle performed with IMFpred. The Area Under the Curve was found to be 92% (0.9176; 95% CI: 0.8141–1.0000; positive likelihood ratio = 16.9749) and using an optimal cut-off value of 4.50 IMF%, sensitivity was 88% and specificity was 90%.

**Figure 4:**
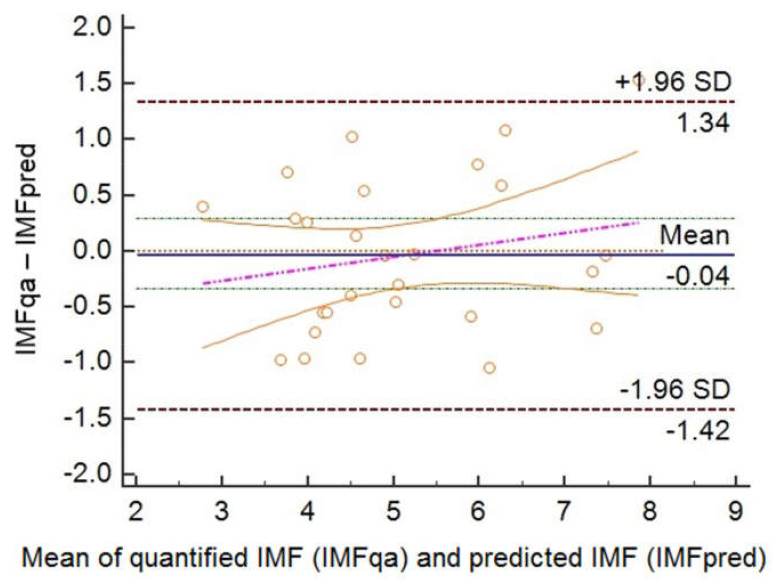
Bland-Altman plot of quantified IMF (IMFqa) versus predicted IMF (IMFpred).

Moreover, a Bland-Altman plot was used to test the agreement between IMFqa and IMFpred (Figure 4). The results from the plot showed an upper limit of +1.34 and a lower limit of −1.42 (±1.96 SD), with a mean of −0.04. All the variables included were statistically significant.

## 4. Discussion

In the present study, seven texture parameters were found to be significant and were included in a model to predict the IMF content in meat samples. With the equation presented here, using only US imaging, the R^2^ was 0.76. First attempts in application of US to carcasses had quite low R^2^-values. They ranged between 0.12 and 0.14 in Liu, et al. [24], and was just slightly greater in Whittaker et al. [16]. Over time, there was an improvement in the US estimation of IMF, and the determination coefficient increased to R^2^ = 0.57 [6]. This is one of the best determination coefficients for IMF% estimation in carcasses with US. The study proposed by Indurain, et al. [6] required gathering of a conspicuous number of parameters. Such method required other variables than US imaging, combining the US measurement of grey level at the 12th rib with thoracic depth and carcass fatness score. The method in the present study used only US parameters and is therefore less labor-intensive and time consuming.

At present, most of the studies have dealt with the estimation of IMF on live animals [17,25,26,27] and only a few on the estimation of IMF after-slaughter. In vivo studies are not only prevalent, but are also characterized with higher R^2^-values. For instance, R^2^-values in in vivo studies ranged from R^2^ = 0.52 to R^2^ = 0.82 [17,25,26,27].

Determination coefficient in the present study (R^2^ = 0.76) could be included in the highest range of all coefficients found in literature (R^2^ ranging between 0.12–0.85) [15,17,18,24,27,28,29]. However, US applied to carcass measures appear to be less reliable than US used in vivo. The method proposed in this study was applied also in Fiore, et al. [18], only on live animals, with a greater determination coefficient (R^2^ = 0.85). Similar results were observed in a study from Whittaker et al. [16] with both in vivo and after slaughter sonograms. Again, R^2^-values from in vivo images were higher than post-mortem ones, even though those images were taken on the same animals, and with the same procedure. Such an outcome could be due to capillary blood loss and tissue change because of the lack of oxygen during slaughter [16]. Capillary blood produces a scattering effect of ultrasonic waves. They hypothesized that, when the animal is slaughtered and blood is drained, the capillaries collapse, reducing the scattering effect of ultrasonic waves [16]. This could apply also to the present study. Moreover, samples in the present study underwent aging in chilling room for seven days before US imaging, which may have caused a loss of water in meat. 

The average IMF content of meat samples in the study was 5.10 ± 1.44%, which is higher than reported by other researchers with values of 1.12% and 3.25% [30,31]. Percentage and distribution of IMF are highly variable depending on nutrition (energy and protein levels), gender, genetic factors (breed), and slaughter weight [32]. The short range of IMF% could be a limitation of the present study. It is possible that application of the presented formula to animals with a different range of IMF% could reduce prediction precision. However, this is a methodological study. We encourage validation of the proposed method on different IMF% and therefore testing on animals of different breeds, sex, age and dietary practices.

Considered all the limitations, the present study had top range R^2^-values. Moreover, Bland-Altman plot depicted in Figure 4 shows a high agreement between IMFqa and IMFpred, and area under the ROC curve (Figure 3) was 0.92. IMFpred seems to have a good diagnostic performance. The proposed procedure seems promising because it is fast and simple in its applicability. It used as few parameters as possible, collected by a single US image and required no other features to enhance reliability. However, further testing is still needed.

We encourage further testing on both different IMF% ranges and with no pre-processing of the meat. Rapidity and easiness of use could make this method a useful tool for meat assessments at the slaughterhouse. Beef grading systems could benefit from a reliable and fast method of estimating IMF in beef as this could lead to a better categorization and economic estimation of the carcasses.

## 5. Conclusions

The present study suggests that objective determination of IMF% in the LT muscle with good accuracy is possible, suggesting a potential use of US imaging in routine beef grading practices. However, this is a methodological study, and the proposed method should be validated on different muscles and on animals of different breed, sex, age and dietary practices, with different IMF% ranges and with no pre-processing of the meat and in a chiller environment. Additionally, further studies on the same animal kind are needed to assess for accuracy and repeatability. If the proposed method is accurate in other settings, US could be used routinely to evaluate IMF in meat. This could lead to more consistent beef products, therefore enhancing consumers’ satisfaction and commercial standardization programs.

## Figures and Tables

**Table 1:** Ingredients and chemical composition of the total mixed ratio used for the heifers in the study.

Feed Ingredients	Total Dry Matter (%)
Maize silage	33.2
Corn mash	13.63
Corn gluten feed	9.14
Maize meal	10.78
Soybean meal 44	2.76
Sugar beet pulps	9.29
Wheat straw	9.2
Protein, vitamin, and mineral premix ^1^	12
**Chemical Composition**	
Dry matter (%)	54.02
Crude protein	13.85
Ether extract	3.29
Ash	5.84
Neutral detergent fiber	37.22
Non-fiber carbohydrates	31.15

^1^ Protein, vitamin, and mineral premix: vitamin A (45,000 IU/kg), vitamin D3 (4500 IU/kg), vitamin E (54 mg/kg), vitamin PP (45 mg/kg), choline (194.60 mg/kg), manganous sulfate (277.20 mg/kg), copper sulfate (141.48 mg/kg), selenium (0.99 mg/kg), zinc sulfate (792 mg/kg), ferrous carbonate (372.60 mg/kg), calcium (5.54 mg/kg), urea (37,240 mg/kg).

**Table 2:** Mean ± standard deviation of the absolute values of the texture parameters included in the regression equation for the three different groups (Group 1: IMF ≤ 4.24%; Group 2: 4.25% ≤ IMF ≤ 5.75%; and Group 3: IMF > 5.76%). The category for each texture parameter is also reported.

Texture Parameter	Texture Category	Group 1	Group 2	Group 3
GrKurtosis	Gradient	2.14 ± 0.35 ^a^	1.38 ± 0.83 ^b^	3.44 ± 1.43 ^a^
Teta2	Autoregressive model	−0.43 ± 0.03 ^a^	−0.47 ± 0.03 ^b^	−0.47 ± 0.04 ^b^
Teta4	Autoregressive model	0.09 ± 0.02	0.10 ± 0.02	0.09 ± 0.03
S(2,2)InvDfMom	Co-occurrence matrix	0.22 ± 0.02	0.23 ± 0.02	0.21 ± 0.02
S(3,−3)Contrast	Co-occurrence matrix	42.13 ± 9.72	40.47 ± 16.46	53.29 ± 20.54
S(4,−4)DifEntrp	Co-occurrence matrix	1.17 ± 0.05	1.15 ± 0.08	1.21 ± 0.08
45dgr_ShrtREmp	Run-length matrix	0.91 ± 0.01	0.90 ± 0.01	0.91 ± 0.01

^a,b^ Statistically significant differences between groups as the result of a Kruskal-Wallis H test with a Bonferroni post-hoc test; GrKurtosis (χ^2^ = 10.867; *p* = 0.004); Teta2 (χ^2^ = 7.112; *p* = 0.029); Teta4 (χ^2^ = 1.195; *p* = 0.550); S(2,2)InvDfMom (χ^2^ = 3.439; *p* = 0.179); S(3,−3)Contrast (χ^2^ = 1.637; *p* = 0.441); S(4,−4)DifEntrp (χ^2^ = 1.532; *p* = 0.465); 45dgr_ShrtREmp (χ^2^ = 3.849; *p* = 0.146).

**Table 3:** Summary of mean, SD, minimum value and maximum value of quantified IMF (IMFqa, extracted from the meat) and of predicted IMF (IMFpred) in the three groups (Group 1: IMF ≤ 4.24%; Group 2: 4.25% ≤ IMF ≤ 5.75%; and Group 3: IMF > 5.76%).

	Group 1		Group 2		Group 3	
	IMFqa	IMFpred	IMFqa	IMFpred	IMFqa	IMFpred
Mean	3.76	4.06	4.99	5.10	7.16	6.71
SD	0.41	0.71	0.40	0.81	0.75	0.91
Maximum value	4.12	5.08	5.61	6.63	8.64	7.71
Minimum value	2.97	2.57	4.31	4.00	6.37	5.59

## Data Availability

The datasets used and/or analysed during the current study are available from the corresponding author on reasonable request.
